# Tenosynovial Giant Cell Tumor After ACL Reconstruction With Autograft

**DOI:** 10.7759/cureus.21829

**Published:** 2022-02-02

**Authors:** Michael Booth, Edward B McDonough

**Affiliations:** 1 Orthopaedics, West Virginia University, Morgantown, USA; 2 Orthopaedic Surgery, West Virginia University, Morgantown, USA

**Keywords:** tenosynovial giant cell, acl, sports, trauma, orthopaedics trauma

## Abstract

A 25-year-old male developed left knee pain several years after anterior cruciate ligament (ACL) reconstruction. MRI showed a suspected cyclops lesion over the anterior portion of the knee. The patient underwent diagnostic knee arthroscopy with lesion removal, and it was discovered the patient had a tenosynovial giant cell tumor. A tenosynovial giant cell tumor is a rare intraarticular lesion that requires a high suspicion for clinical diagnosis. Management is currently centered around arthroscopic versus open removal of the lesion with serial monitoring and advanced imaging for recurrence.

## Introduction

Synovial giant cell tumor most frequently occurs on the hand and uncommonly on the ankle or knee [1]. Tenosynovial giant cell tumor (TSGCT) is a family of lesions involving the joint synovia, bursae, and tendon sheath [2]. TSGCT is considered rare and has an incidence of 43 cases per million population per year and typically affects young patients in the third and fourth decades [3]. Localized TSGCT grows slowly over time and patients tend to present with pain and effusion in the joint and sometimes mechanical symptoms such as catching and locking [3]. There have been several case reports citing intraarticular knee TSGCT but there have been no previous reports of a TSGCT after anterior cruciate ligament (ACL) reconstruction.

## Case presentation

A 25-year-old male presented to our office complaining of intermittent left knee pain and swelling after undergoing a left knee ACL reconstruction with hamstring autograft six years ago. He injured his knee while performing a field goal in a collegiate football game and heard a "pop" with subsequent knee-buckling and swelling. The patient had an unremarkable postoperative course after his surgery and was very satisfied with his knee up until about the last month prior to presentation. The patient does not have any medical problems, has no family history of cancer, is not taking any medications, and enjoys jogging and playing basketball.

**Figure 1:**
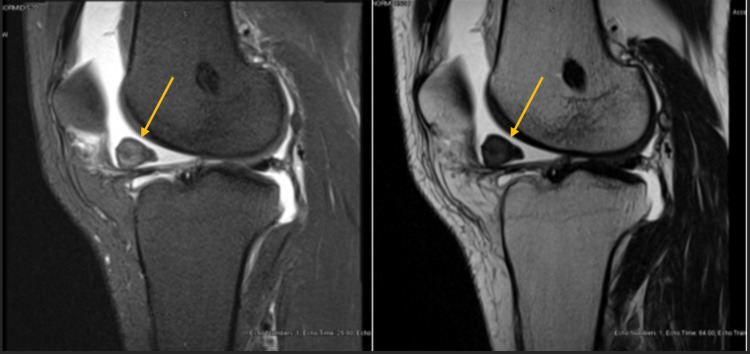
T2 (left) and T1 (right) MRI sagittal images of the left knee showing a heterogeneous foreign lesion over the anterior portion of the knee

On physical exam, the patient had a left knee effusion when compared to his contralateral side, the range of motion of his left knee was from 0-120 degrees, with negative Lachman test and negative anterior/posterior drawer, and his knee was stable at 0 and 30 degrees of flexion to varus and valgus stress. The patient had an MRI performed in the emergency department several days prior, which showed a 17 mm x 26 mm x 16 mm lesion in the anterior portion of the left knee near the infrapatellar fat pad, which was read as a cyclops lesion (Figure 1). Given his mechanical symptoms and the large size of the lesion, the patient underwent a left knee diagnostic arthroscopy with foreign body removal.

**Figure 2:**
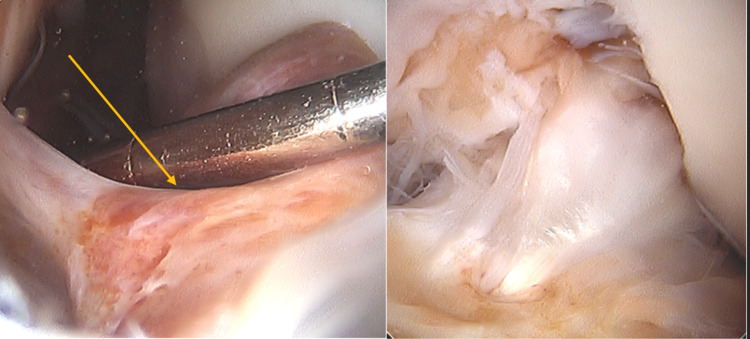
Intraoperative images of the knee showing the tenosynovial giant cell tumor before (left) and after (right) debridement, which revealed the ACL ACL: anterior cruciate ligament

**Figure 3:**
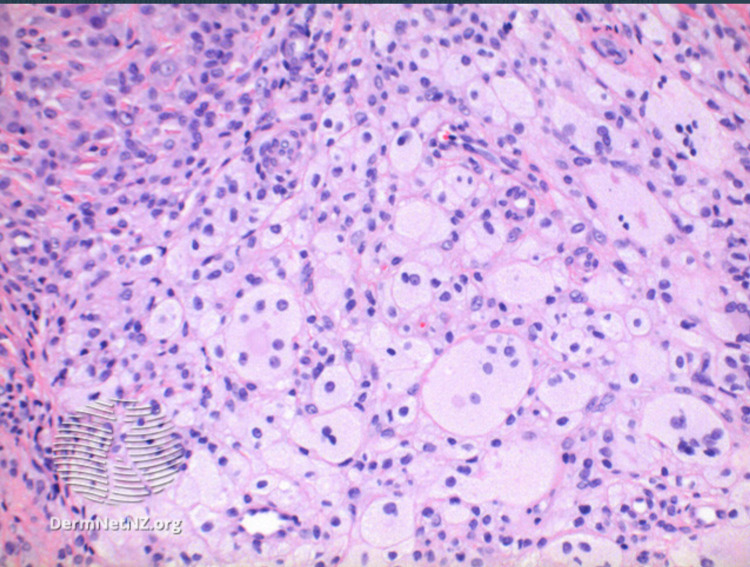
Giant cell tumor of tendon sheath pathology Image used in accordance with DermNet NZ image use policy. Link: https://dermnetnz.org/topics/giant-cell-tumour-of-tendon-sheath-pathology

Intraoperatively, there was no evidence of ACL tear or fraying, and the medial and lateral menisci were intact. The infrapatellar fat pad was noted to be hypertrophied and there was a large friable loose body with a stalk off of the previous ACL graft located within the notch (Figure 2), which was removed with a grasper and sent for pathology. The final pathology the next day revealed a localized type tenosynovial giant cell tumor (Figure 3). After consulting with our orthopedic oncology team, our plan is to monitor the left knee for recurrence with MRI at six months and again in two years' time.

## Discussion

There have been previous case studies reporting a localized tenosynovial giant cell tumor surrounding the Hoffa fat pad but never after an ACL reconstruction [1,4]. The initial lesion for our patient was read as a cyclops lesion on MRI but there can be subtle differences noted. MRI for a TSGCT shows a well-circumscribed heterogeneous lesion on both T1 and T2 MRI due to the hemosiderin pigment content in the lesion [5]. A tenosynovial giant cell tumor is a rare intraarticular lesion that requires a high suspicion for clinical diagnosis. Management is currently centered around arthroscopic versus open removal of the lesion with serial monitoring and advanced imaging for recurrence [5].

The causes of TSGCT may include benign neoplastic process or reaction to an unknown stimulus versus response to repeated episodes of trauma or hemarthrosis [6]. In localized forms of TSGCT, the recurrence rate in both open and arthroscopic excision of these lesions in the knee is 7.1% [7]. There is currently insufficient evidence to support open versus arthroscopic removal of the lesion. Open synovectomy entails a greater risk of knee stiffness and longer hospital stay and rehabilitation [8-9]. Arthroscopy, on the other hand, only enables partial resection, with an elevated risk of recurrence [10]. There are certain measures, such as limiting portal sites, excising the portal site tracts, and excising the lesion with the shaver one high suction, which may help reduce the rate of recurrence.

There have not been formal randomized studies comparing open surgery versus arthroscopic surgery. Radiation therapy has been used as an adjunct treatment to surgery particularly in cases of incomplete resection of tumor [11]. There have been limited studies as to the long-term consequences of intraarticular radiation and the possibility of radiation-induced sarcoma [12]. The Food and Drug Administration (FDA) approved a CSF-1 inhibitor, pexidartinib, for the treatment of adult patients with TSGCT. Investigational therapies include modulation of the CSF1R receptors, which addresses the underlying problem of the over-expression of CSF1 [13].

## Conclusions

Overall, a localized tenosynovial giant cell tumor is a rare intraarticular lesion that requires a high suspicion for clinical diagnosis. Management is currently centered around arthroscopic versus open removal of the lesion with serial monitoring and advanced imaging for recurrence. The Diagnosis of this lesion in such an uncharacteristic location requires critical evaluation of advanced imaging as well as a pathologist available to evaluate the specimen. This unusual case in a young patient will also require routine evaluation for potential recurrence. The patient was followed at two weeks, six weeks, and three months postoperative as part of the routine ACL postoperative protocol. We plan to MRI the knee again at six months postoperative to monitor for recurrence.
